# Periodontitis and stroke: A Mendelian randomization study

**DOI:** 10.1002/brb3.2888

**Published:** 2023-01-09

**Authors:** Chaoyang Ma, Min Wu, Jie Gao, Chuanzi Liu, Yi Xie, Qiushi Lv, Xiaohao Zhang

**Affiliations:** ^1^ Department of Endodontology Affiliated Stomatology Hospital of Guangzhou Medical University, Guangzhou Key Laboratory of Basic and Applied Research of Oral Regenerative Medicine Guangzhou China; ^2^ Department of Neurology Jinling Hospital Nanjing Medical University Nanjing China; ^3^ Department of Neurology Jinling Hospital Medical School of Nanjing University Nanjing China; ^4^ Hospital of Stomatology, Guangdong Provincial Key Laboratory of Stomatology, Institute of Stomatological Research, Guanghua School of Stomatology Sun Yat‐sen University Guangzhou China; ^5^ Department of Neurology Nanjing First Hospital Nanjing Medical University Nanjing China

**Keywords:** causal effect, ischemic stroke, Mendelian randomization study, periodontitis

## Abstract

**Background and purpose:**

Periodontitis has been implicated in the incidence of ischemic stroke. However, the generalizability of results to individuals with different subtypes of periodontitis is unknown. We aimed to investigate the causal relationship of chronic periodontitis (CP) and aggressive periodontitis (AgP) with ischemic stroke and its subtypes in the Mendelian randomization framework.

**Methods:**

The genetic proxies of CP were derived from large‐scale summary statistics from the UK Biobank datasets (950 cases and 455,398 controls). The genetic associations of AgP were selected from another large genome‐wide association study of European ancestry (851 cases and 6836 controls). The instruments of ischemic stroke (34,217 cases and 406,111 controls) and its subtypes were selected from the MEGASTROKE consortium of European ancestry. The inverse variant weighted method was performed to determine the causal inference and a comprehensive set of sensitivity analyses to test the robustness of the results.

**Results:**

In population‐wide genetic analysis, there was no association of genetically predicted AgP (odds ratio [OR], 0.982; 95% confidence interval [CI], 0.956–1.009; *p* = .197) with ischemic stroke or its subtypes. For patients with CP, there was also no significant causal inference on ischemic stroke (OR, 1.017; 95% CI, 0.992–1.043; *p* = .184). However, regarding the stroke subtypes, the genetic analysis provided evidence of a causal relationship of CP with cardioembolic stroke (OR, 1.052; 95% CI, 1.002–1.104; *p* = .042), but not with large artery atherosclerosis (OR, 1.005; 95% CI, 0.944–1.069; *p* = .875) or small vessel occlusion (OR, 1.039; 95% CI, 0.981–1.101; *p* = .193).

**Conclusion:**

This study suggested that there was a potential causal effect of CP on cardioembolic stroke.

## Graphical abstract

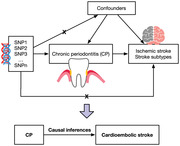

## 1 INTRODUCTION

Periodontitis represents a chronic inflammation status, caused by the pathogenic microflora in the dental plaque that leads to irreversible destruction of the connective tissue of the tooth and alveolar bone^1^. The prevalence of periodontitis is high, approximately accounting for 90% of the worldwide population (Pihlstrom et al., 2005), and it can be classified as aggressive periodontitis (AgP) and well‐known chronic periodontitis (CP) (Tonetti et al., 2018). AgP is a severe type of periodontitis with an early onset and a higher rate of progression (Susin et al., 2014), while CP is more prevalent in the old population and may not progress for 5–6 years in 85% of patients (Renvert et al., 1990).

Some observational studies have suggested that periodontitis is positively associated with the risk of cardiovascular diseases (Destefano et al., 1993), possibly mediated by inflammatory mechanisms (You et al., 2009). However, reports on the impact of periodontitis on ischemic stroke are inconclusive. A large observational study with a median follow‐up of 12 years indicated that the risk of ischemic stroke was approximately 1.6 times high in patients with periodontitis and fewer teeth (Joshipura et al., 2003). In addition, some studies have reported that periodontitis is an independent predictor of ischemic stroke (Grau et al., 2004; Sen et al., 2018), while others reported controversial results (Zhou et al., 2021). Meanwhile, the generalizability of causal inference to individuals with different subtypes of periodontitis is unknown, and it is indeed of great significance to clarify the association of periodontitis subtypes with ischemic stroke.

Mendelian randomization (MR) is a method to infer causal inference using genetic proxies that are specifically related to a particular exposure (i.e., CP). It is an alternative approach for a randomized trial because of the segregation of alleles at conception, which means the genetically defined population cannot be affected by confounding and reverse causation (Thanassoulis & O'donnell, 2009). Therefore, we aimed to perform the two‐sample Mendelian randomization study to investigate the causal inference of CP and AgP on ischemic stroke and its subtypes.

## 2 METHODS

### 2.1 Selection of the genetic instruments

The genetic proxies of CP were derived from large‐scale summary statistics of European individuals from the UK Biobank dataset (950 cases and 455,398 controls) (Jiang et al., 2021). The UK Biobank is a prospective study of approximately 500,000 population aged 40–69 years at baseline and launched from 2006 to 2010 with a median follow‐up of 10.9 years (Sudlow et al., 2015). In addition, the genetic instruments of AgP were derived from another large genome‐wide association study of European ancestry (851 cases and 6836 controls, replicated in 223 cases and 564 controls) (Munz et al., 2017). As a minuscule number of single nucleotide polymorphisms (SNPs) associated with AgP or CP were left at the level of *p*‐value < 5 × 10^−8^, we selected the genetic instruments with *p*‐value < 1× 10^−5^. These SNPs were clumped for independence (*R* ^2^ cut‐off of 0.001, distance window, 10,000 kb) based on the European sample of 1000 genomes data (Abecasis et al., 2010).

### 2.2 Outcome data sources

The primary outcome of this study is ischemic stroke (34,217 cases and 406,111 controls). Moreover, we performed an exploratory analysis for the causal relationships of CP and AgP with different ischemic stroke subtypes, including large artery atherosclerosis (LAA) (4373 cases and 146,392 controls), small vessel occlusion (SVO) (5386 cases and 192,662 controls), and cardioembolic (CE) stroke (7193 cases and 204,570 controls), classified according to the Trial of Org 10172 in acute stroke treatment classification (Adams et al., 1993). All the outcome datasets were derived from the Multiancestry Genome‐wide Association Study of Stroke (MEGASTROKE) consortium in European ancestry individuals (Malik et al., 2018).

### 2.3 Statistical analysis

The fixed‐effects inverse‐variance weighted (IVW) method was performed to obtain the primary MR estimation. In the case of the presence of horizontal pleiotropy in the SNPs, MR‐Egger regression (Bowden et al., 2015) and weighted median (Bowden et al., 2016) were also conducted to test the robustness of the results. In the sensitivity analysis, IVW methods with Cochran's *Q* statistics and MR‐Egger intercept were performed to evaluate the heterogeneity and pleiotropy of individual SNPs, respectively. In addition, a leave‐one‐out analysis was performed to evaluate the robustness of MR results through any outlier SNP.

All statistical analyses were undertaken using the TwoSampleMR package in R statistical software, version 4.1.2. (R Foundation, Vienna, Austria), and a two‐tailed *p*‐value < .05 was considered statistically significant.

## 3 RESULTS

### 3.1 The causal effect of CP on ischemic stroke

**FIGURE 1:**
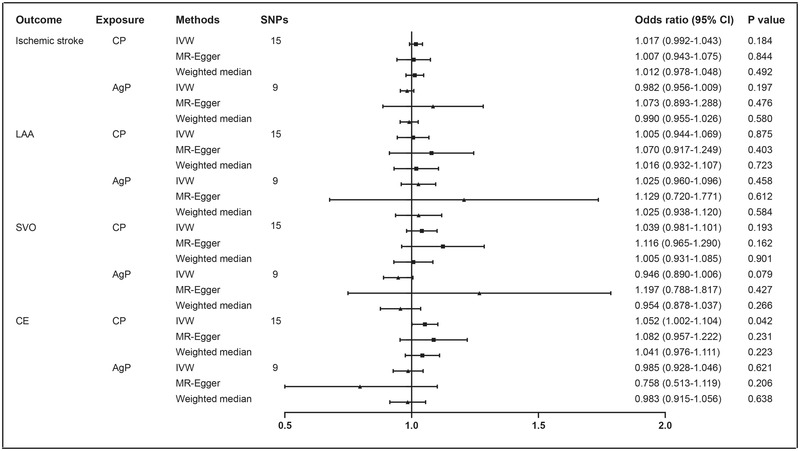
Mendelian randomization analysis results for periodontitis and ischemic stroke. Abbreviations: AgP, aggressive periodontitis; CE, cardioembolic; CI, confidence interval; CP, chronic periodontitis; IVW, inverse‐variance weighted; LAA, large‐artery atherosclerosis; SNPs, single nucleotide polymorphisms; SVO, small‐vessel occlusion

After harmonizing the data of CP with ischemic stroke and its subtypes, we obtained 15 SNPs for CP as genetic instruments. In the overall IVW analysis, there was no causal inference of CP on ischemic stroke (odds ratio [OR], 1.017; 95% confidence interval [CI], 0.992–1.043; *p* = .184), as well as in the MR‐Egger regression and weighted median methods (Figure 1 and Figure S1).

In the exploratory analysis of ischemic stroke subtypes, the positive causal inference of CP on CE stroke was observed in the IVW method (OR, 1.052; 95% CI, 1.002–1.104; *p* = .042), and a similar pattern of effect sizes was yielded in the MR‐Egger regression and weighted median methods, despite a lower statistical power (Figure 1 and Figure S1). In the sensitivity analysis, there was no heterogeneity (*p* for Cochran's Q, 0.916) or pleiotropy (*p* for MR‐Egger intercept, 0.632) among these SNPs (Table S1). Meanwhile, there were also no potential outlier SNPs in the leave‐one‐out analysis in the associations of CP with CE stroke, which confirmed the robustness of the results (Figure S2). The contributions of individual SNPs and overall estimated effects were shown in Figure S3.

However, there was no significant inference of CP on LAA (OR, 1.005; 95% CI, 0.944–1.069; *p* = .875) or SVO (OR, 1.039; 0.981‐1.101; *p* = .193) (Figure 1 and Figure S1).

### 3.2 The causal effect of AgP on ischemic stroke

The nine SNPs related to AgP were selected as the genetic proxies in ischemic stroke and its subtypes participants. In the primary analysis, none of the nine individual SNPs were associated with the incidence of ischemic stroke (OR, 0.982; 95% CI, 0.956–1.009; *p* = .197). In addition, the associations between genetically predicted AgP and stroke subtypes were also negative (Figure 1 and Figure S1). No potential heterogeneity or pleiotropy was found among these proxies in the sensitivity analysis (Table S1). A comprehensive set of sensitivity analyses confirmed the robustness of the results (Figures S4–S6).

## 4 DISCUSSION

This MR study showed that there was a causal inference of genetically defined CP on CE subtypes but was unable to provide evidence for the causal relationship of AgP with ischemic stroke and its subtype.

The finding from this study was consistent with several observational studies showing a positive association between periodontitis and ischemic stroke (Grau et al., 2004; Sen et al., 2018). A meta‐analysis of nine cohort studies has reported that periodontitis has increased the risk of ischemic stroke by approximately 1.53 folds (Lafon et al., 2014). In addition, Souvik et al. (2001) have reported that periodontitis is an independent risk factor for ischemic stroke in a large cohort study with a follow‐up of over 15 years. In that study, the risk of ischemic stroke mediated by the CE mechanism in patients with periodontitis was approximately 2.6 folds higher than their counterparts, while it only confirms the correlation, not the causality between these two traits. Meanwhile, it should be noted that some limitations, including different definitions of periodontitis, broadly selected groups of populations, and some confounders, like socioeconomic status and lifestyle are common in conventional observational studies, all of which may affect the interpretation of results. However, some studies have yielded controversial results (Zhou et al., 2021). In a previous study (Zhou et al., 2021), periodontitis was found not to be associated with the incidence of ischemic stroke, while this study did not classify periodontitis into different subtypes that may affect the explanation for the relationship between these two traits exactly. In our MR study, periodontitis was categorized into CP and AgP, and we found a causal relationship between CP and CE stroke based on large‐scale GWAS summary statistics, which are less susceptible to the confounders and reverse causation.

The underlying mechanisms of CP on CE stroke are not well elucidated. Inflammation may be one of the potential mechanisms that mediated this association, as CP represents an inflammation status induced by the interplay between bacterial infection and host immune response (Kinane, 2001), usually with elevated inflammatory biomarkers (Loos, 2005). Several studies suggested that patients affected by the CE had relatively elevated proinflammatory biomarkers, like C‐reactive protein, interleukin‐6, and interleukin‐1 (Licata et al., 2009; Tuttolomondo et al., 2012, 2009). Moreover, these proinflammatory biomarkers also have an important role in the initiation of atrial fibrillation (AF) by affecting the electrophysiology and structural properties of the atrial (Hu et al., 2015). Findings from a large biracial cohort study showed that periodontitis was significantly associated with AF (Sen et al., 2021). In that study, AF was found to be a mediator of the association between periodontitis and CE stroke.

In addition, previous studies have reported that periodontitis was also related to the processes of atherosclerosis and endothelial dysfunction, as some odontogenic bacteria like *Porphyromonas gingivalis, Fusobacterium nucleatum*, and *Actinobacillus actinomycetemcomitans* were found to colonize in the atherosclerotic plaque (Ford et al., 2005; Haraszthy et al., 2000). This pathogens‐derived inflammation increased the risk of cardiovascular diseases and ischemic stroke. However, the causal relationships of CP between ischemic stroke mediated by the LAA or SVO mechanism were not demonstrated in our study, while it does not mean that there was no association among these traits. Reversely, the causal relationships of CP with LAA or SVO‐related stroke should be portrayed thoroughly in further studies.

Moreover, our study did not find any causal effect of AgP on ischemic stroke and its subtypes. AgP is a severe type of periodontitis with rapid attachment loss and resorption of alveolar bone (Susin et al., 2014). Schenkein et al. (2007) have reported that patients with AgP had relatively elevated anti‐cardiolipin and serum adhesion molecule levels, which are associated with the incidence of myocardial infarction and stroke. But the association between AgP and ischemic stroke might be less prominent than in patients with CP, regarding the more acute and severe nature of AgP, especially it often occurs in young people who were more likely to focus on dental care. However, the causal inference of AgP on ischemic stroke also needs further investigation.

In the present study, we determined a causal relationship between CP and CE stroke, which may be vital for improving stroke prevention. Strengths of this MR study include large‐scale GWAS cases and the MR design, by which the effect sizes may not be affected by reverse causation, recall bias, as well as some unknown confounders. Moreover, a comprehensive set of analyses increased the robustness of our results. However, there are also some potential limitations. First, the genetic instruments in this study were derived from participants of European ancestry, so the generalizability of results to other populations may be confined by the genotype–environment interactions. Second, unaddressed biases may exist as the exposures and outcome were derived from different datasets, although there was no potential pleiotropy or heterogeneity in the sensitivity analysis. Third, although the causal relationship of CP with CE stroke was established in this MR study, whether to perform preventive treatments for ischemic stroke in the different stratified severity of CP patients warrants further research.

## 5 CONCLUSION

This study suggested that there was a potential causal effect of CP on cardioembolic stroke.

## AUTHOR CONTRIBUTIONS

Chaoyang Ma and Xiaohao Zhang designed the study. Chaoyang Ma, Min Wu, and Jie Gao interpreted data and wrote the manuscript. Chuanzi Liu and Yi Xie prepared the tables and figures. Chaoyang Ma and Min Wu were involved in the statistical analyses. Qiushi Lv and Xiaohao Zhang supervised the study. All authors have made an intellectual contribution to the manuscript and approved the submission.

## CONFLICTS OF INTEREST

The authors declare no conflict of interest.

### PEER REVIEW

The peer review history for this article is available at https://publons.com/publon/10.1002/brb3.2888.

## Supporting information


**Supplemental Table 1**. Heterogeneity and pleiotropy of individual single nucleotide polymorphisms for Mendelian randomization.
**Supplemental Figure 1**. Scatter plot of Mendelian randomization analyses for the causal relationship between chronic periodontitis and ischemic stroke.
**Supplemental Figure 2**. Leave‐one‐out analysis results for the causal effect of non‐chronic periodontitis on the risk of ischemic stroke.
**Supplemental Figure 3**. The contribution of individual SNPs and overall estimated effects of chronic periodontitis.
**Supplemental Figure 4**. Scatter plot of Mendelian randomization analyses for the causal relationship between aggressive periodontitis and ischemic stroke.
**Supplemental Figure 5**. Leave‐one‐out analysis results for the causal effect of non‐aggressive periodontitis on the risk of ischemic stroke.
**Supplemental Figure 6**. The contribution of individual SNPs and overall estimated effects of aggressive periodontitis.

## Data Availability

The datasets analyzed in this study are publicly available summary statistics.
