# Supplementary material for: Periodontitis and stroke: A Mendelian randomization study
Source: Brain Behav. 2023 Jan 9;13(2):e2888. doi: 10.1002/brb3.2888 (PMC9927832; doi:10.1002/brb3.2888)
Supplement: Supplementary file 1 — Supplemental Table 1. Heterogeneity and pleiotropy of individual single nucleotide polymorphisms for Mendelian randomization. Supplemental Figure 1. Scatter plot of Mendelian randomization analyses for the causal relationship between chronic periodontitis and ischemic stroke. Supplemental Figure 2. Leave‐one‐out analysis results for the causal effect of non‐chronic periodontitis on the risk of ischemic stroke. Supplemental Figure 3. The contribution of individual SNPs and overall estimated effects of chronic periodontitis. Supplemental Figure 4. Scatter plot of Mendelian randomization analyses for the causal relationship between aggressive periodontitis and ischemic stroke. Supplemental Figure 5. Leave‐one‐out analysis results for the causal effect of non‐aggressive periodontitis on the risk of ischemic stroke. Supplemental Figure 6. The contribution of individual SNPs and overall estimated effects of aggressive periodontitis. [file BRB3-13-e2888-s001.docx]

**Supplemental Table 1**. Heterogeneity and pleiotropy of individual single nucleotide polymorphisms for Mendelian randomization.

|  |  | **Heterogeneity** |  |  | **P****leiotropy** |  |
| --- | --- | --- | --- | --- | --- | --- |
| **Outcome** | **Exposure** | **Cochran’s Q statistic (IVW)** | ***P* value** |  | **MR-Egger intercept** | ***P* value** |
| Ischemic stroke | CP | 13.85 | 0.461 |  | 0.003 | 0.744 |
|  | AgP | 8.07 | 0.427 |  | -0.025 | 0.372 |
| LAA | CP | 12.43 | 0.572 |  | -0.022 | 0.397 |
|  | AgP | 4.30 | 0.829 |  | -0.027 | 0.683 |
| SVO | CP | 9.48 | 0.799 |  | -0.024 | 0.312 |
|  | AgP | 4.65 | 0.794 |  | -0.066 | 0.302 |
| CE | CP | 7.45 | 0.916 |  | -0.009 | 0.632 |
|  | AgP | 11.10 | 0.196 |  | 0.073 | 0.225 |

Abbreviations: IVW, inverse-variance weighted; CP, chronic periodontitis; AgP, aggressive periodontitis; LAA, large-artery atherosclerosis; SVO, small-vessel occlusion; CE, cardioembolic.

**Supplemental Figure 1.** Scatter plot of Mendelian randomization analyses for the causal relationship between chronic periodontitis and ischemic stroke.


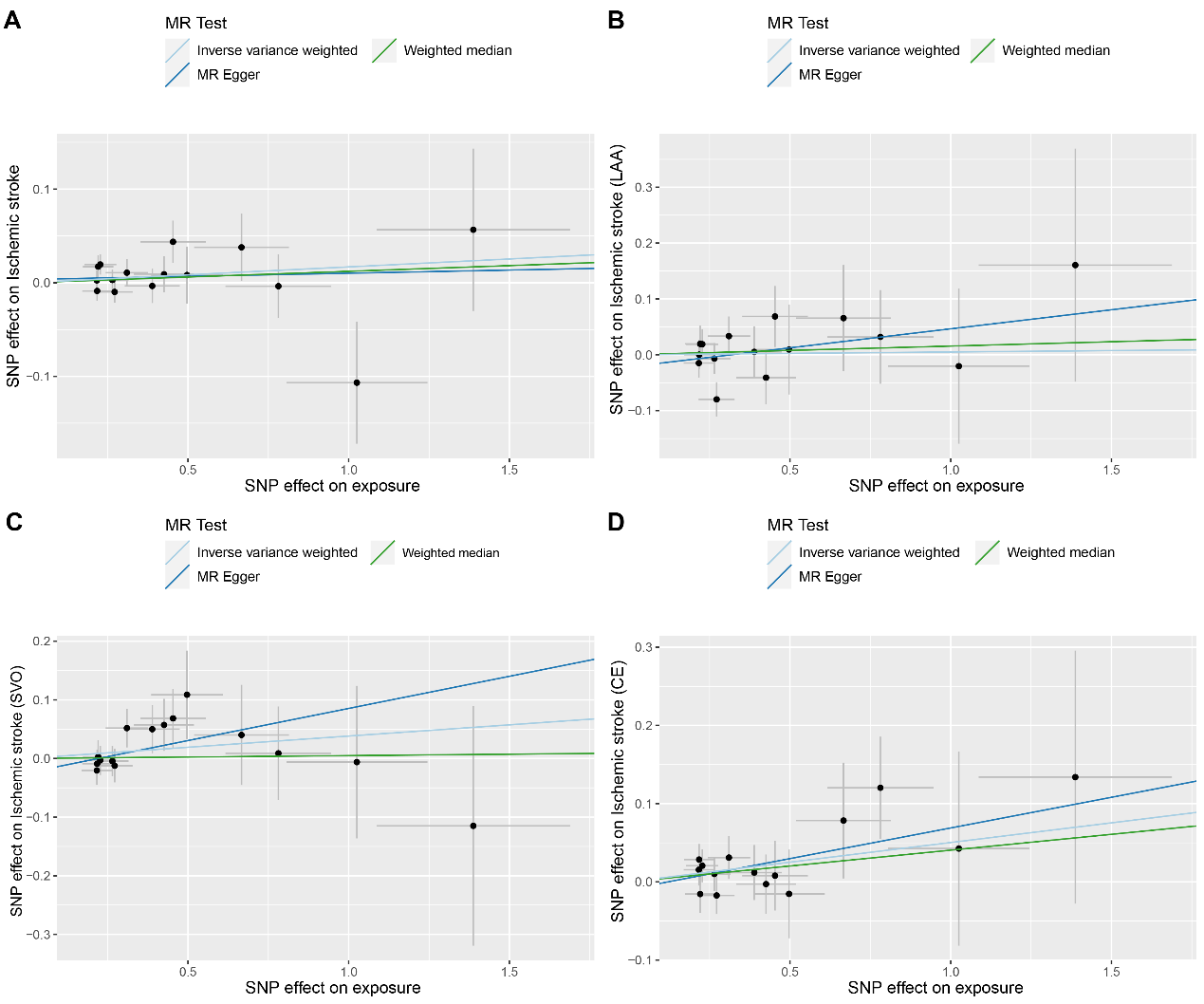


Abbreviations: A. Ischemic stroke; B. Large-artery atherosclerosis; C. Small-vessel occlusion; D. Cardioembolic stroke

**Supplemental Figure 2.** Leave-one-out analysis results for the causal effect of non-chronic periodontitis on the risk of ischemic stroke.


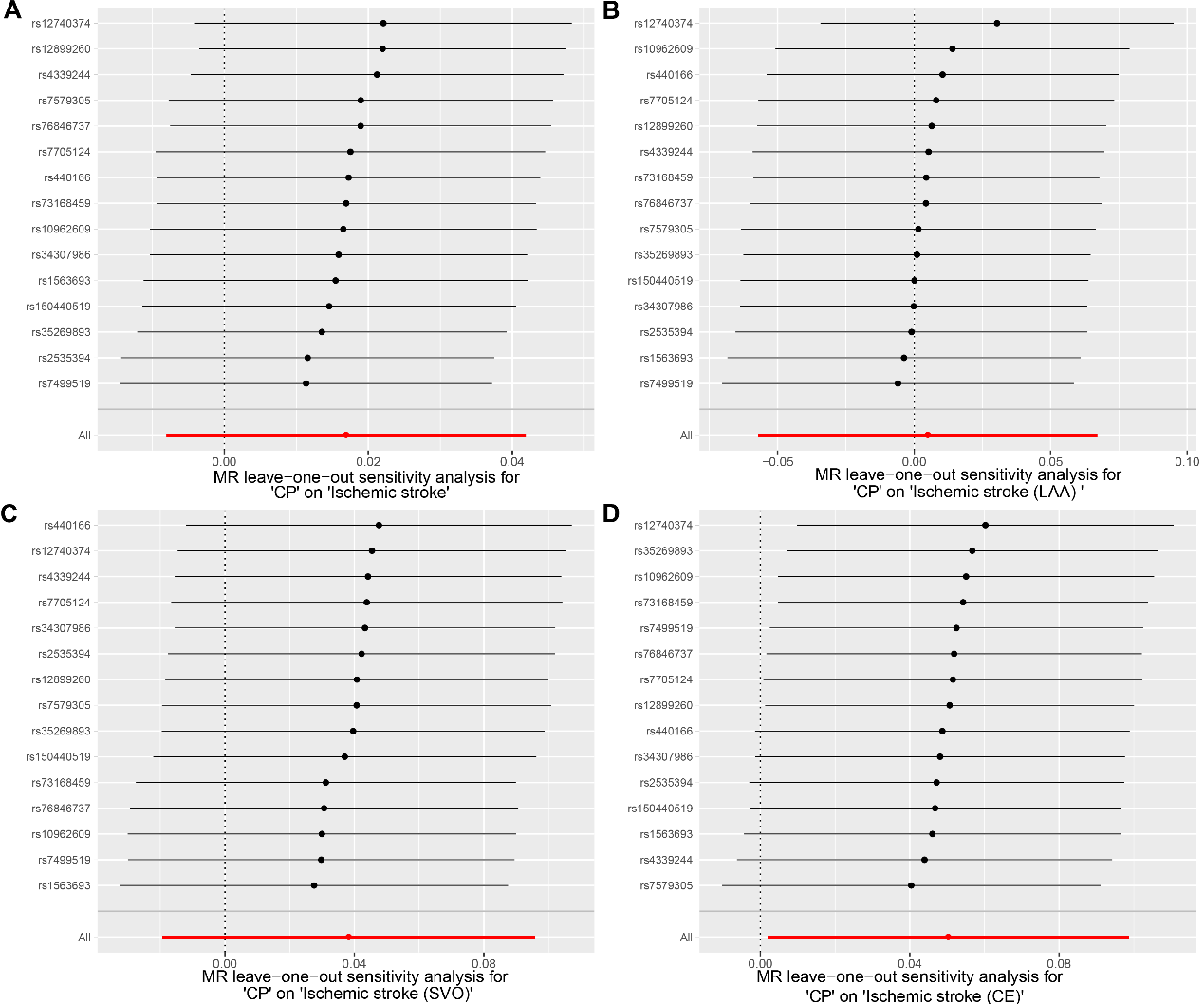


Abbreviations: CP, chronic periodontitis; A. Ischemic stroke; B. Large-artery atherosclerosis; C. Small-vessel occlusion; D. Cardioembolic stroke

**Supplemental Figure 3.** The contribution of individual SNPs and overall estimated effects of chronic periodontitis.


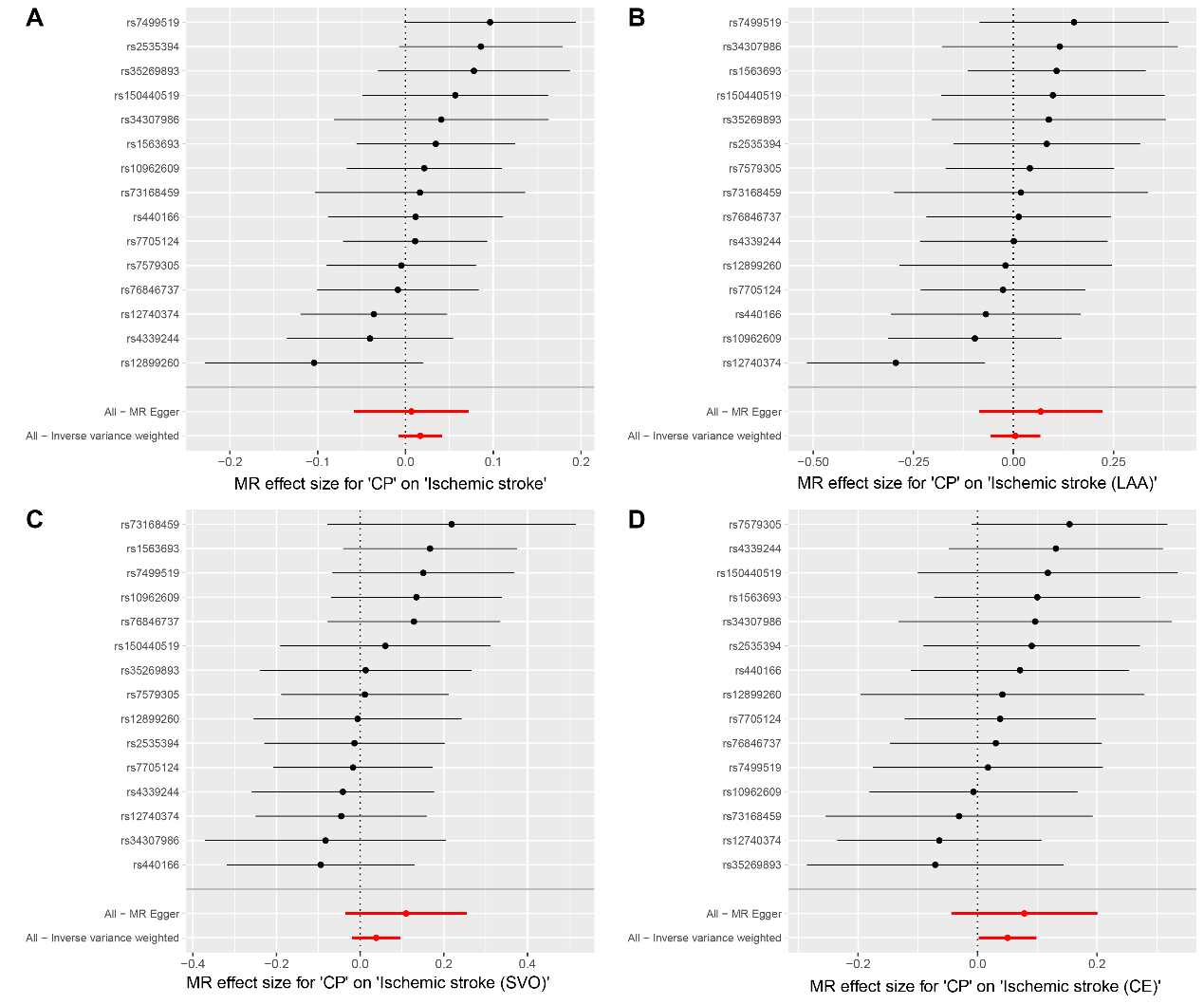


Abbreviations: CP, chronic periodontitis; A. Ischemic stroke; B. Large-artery atherosclerosis; C. Small-vessel occlusion; D. Cardioembolic stroke

**Supplemental Figure 4**. Scatter plot of Mendelian randomization analyses for the causal relationship between aggressive periodontitis and ischemic stroke.


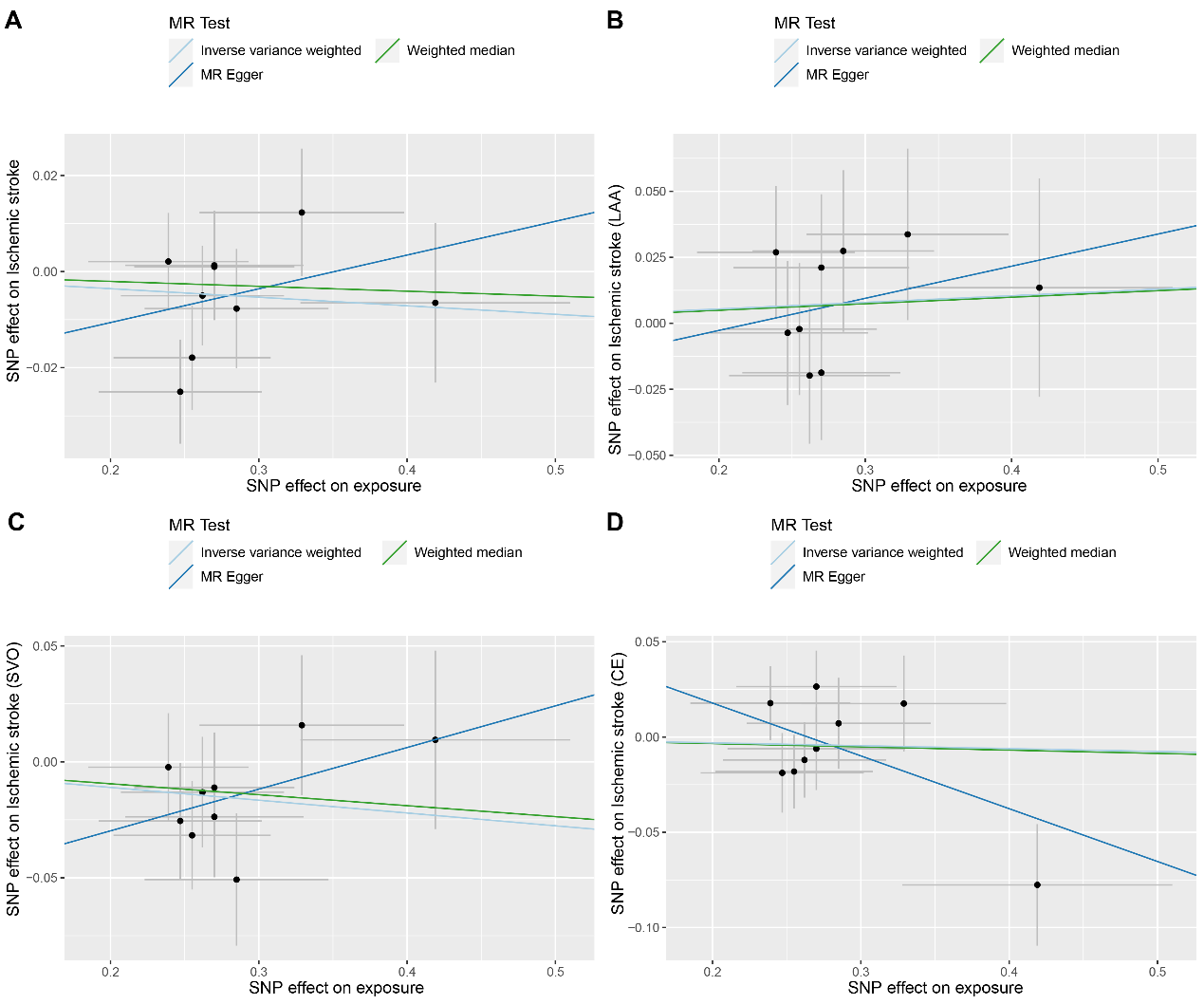


Abbreviations: A. Ischemic stroke; B. Large-artery atherosclerosis; C. Small-vessel occlusion; D. Cardioembolic stroke

**Supplemental Figure 5.** Leave-one-out analysis results for the causal effect of non-aggressive periodontitis on the risk of ischemic stroke.


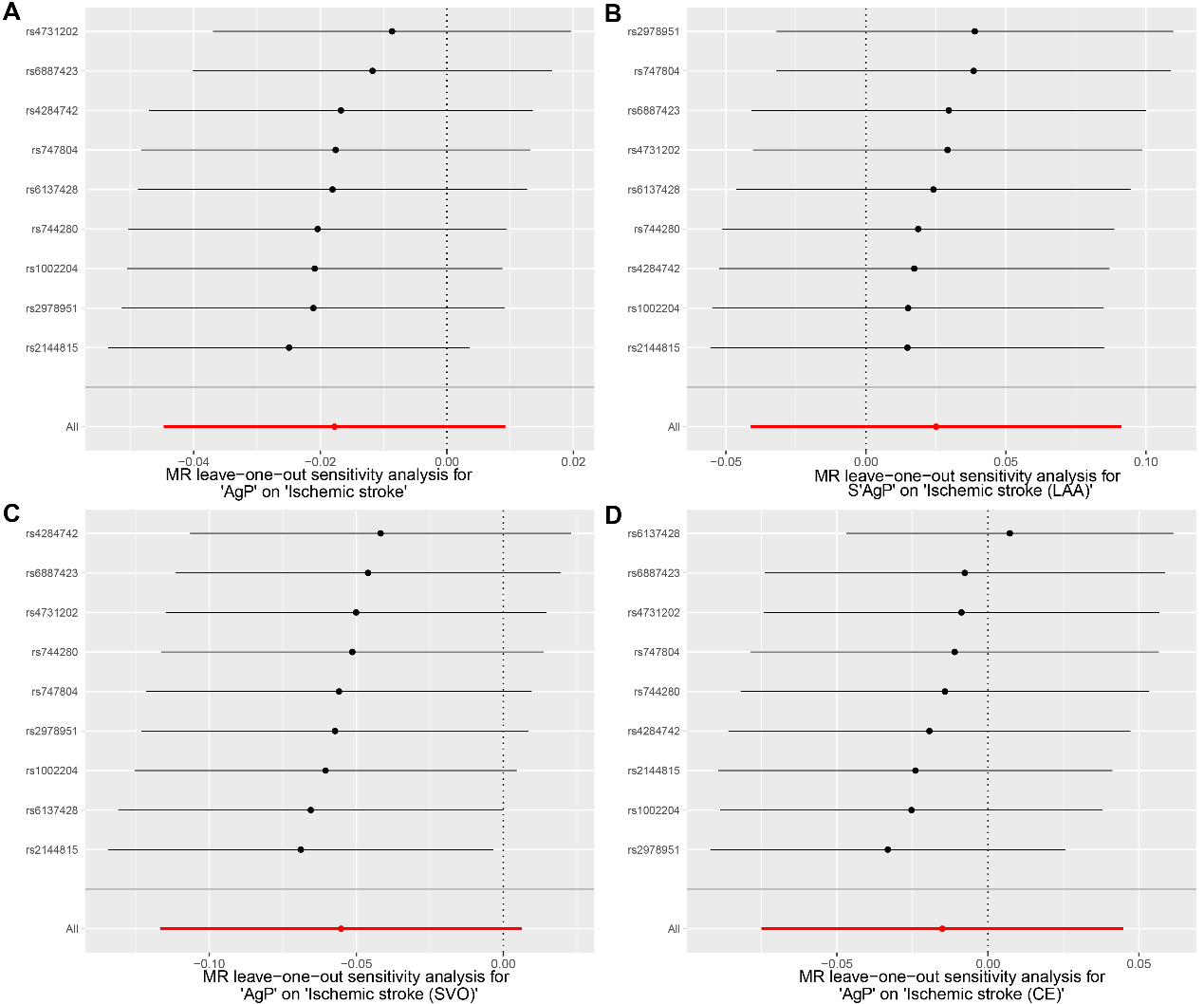


Abbreviations: AgP, aggressive periodontitis; A. Ischemic stroke; B. Large-artery atherosclerosis; C. Small-vessel occlusion; D. Cardioembolic stroke

**Supplemental Figure 6.** The contribution of individual SNPs and overall estimated effects of aggressive periodontitis.


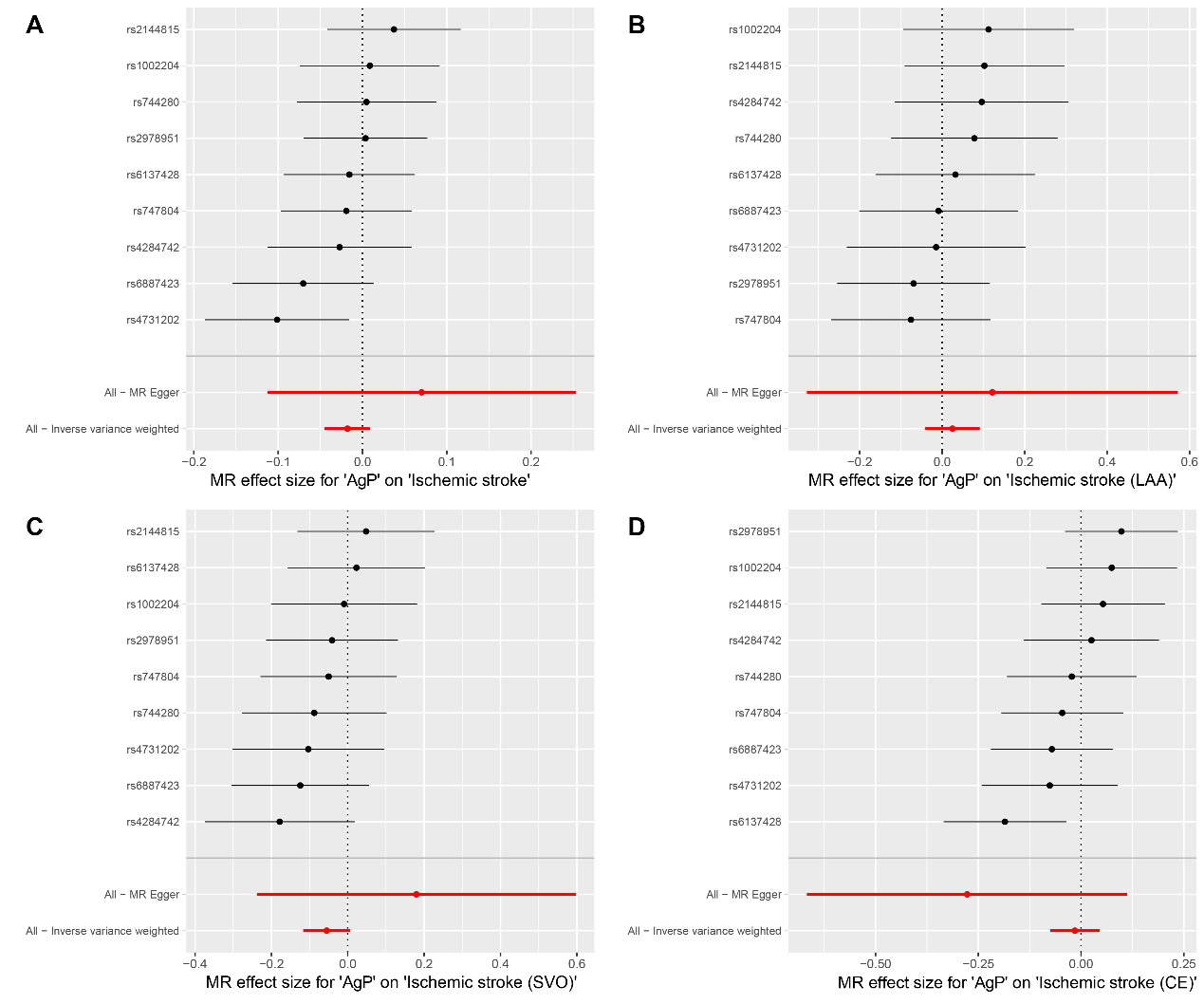


Abbreviations: AgP, aggressive periodontitis; A. Ischemic stroke; B. Large-artery atherosclerosis; C. Small-vessel occlusion; D. Cardioembolic stroke
